# Adverse Effects and Patient Perceptions of Unregulated Arthritis Supplements: A Retrospective Mixed-Methods Study at a Safety-Net Primary Care Clinic

**DOI:** 10.7759/cureus.88378

**Published:** 2025-07-20

**Authors:** Vi D Nguyen, Elizabeth M Canales, Kenneth Pettersen, Arash Nafisi

**Affiliations:** 1 Department of Medicine, University of California Los Angeles David Geffen School of Medicine, Los Angeles, USA; 2 Department of Medicine, Olive View – University of California Los Angeles Medical Center, Los Angeles, USA

**Keywords:** adrenal insufficiency, arthritis, artri king, chronic pain, complementary and alternative medicine, glucocorticoid, medication reconciliation, supplement, supplement use

## Abstract

Introduction

The use of complementary and alternative medicine is prevalent among individuals living with chronic pain. Artri King and similarly branded over-the-counter supplements are advertised as natural arthritis pain relievers but have been found to contain hidden prescription drugs including dexamethasone, diclofenac, and methocarbamol. These unbranded pharmaceutical agents may pose significant health risks, particularly when used without clinical oversight or in combination with other therapies. This study sought to understand patient experiences, perceptions, and motivations for using these supplements despite FDA warnings and the associated potential for harm.

Materials and methods

Between April 2024 and March 2025, clinicians at an urban, academic safety-net primary care clinic in California identified patients who reported using over-the-counter arthritis pain supplements. Clinical data were extracted from the electronic health record. Semi-structured phone interviews were conducted to assess patient-reported motivations, product understanding, duration, dosage, quantity of use, symptom relief, and side effects.

Results

Clinicians identified 14 patients taking arthritis pain supplements reported by the FDA as containing undisclosed drugs (Artri King, Artri Ajo King, RM Flex); 12 of these patients completed phone interviews for this study. Motivations for supplement use included word-of-mouth recommendations, inadequate pain relief from prescribed treatments, and the belief that the supplements were “natural” and therefore safe. All patients reported significant pain relief. None were aware of the presence of hidden ingredients or the potential for adverse effects. Over half of the patients reported side effects. Of the five patients who underwent morning cortisol testing, three had levels consistent with adrenal insufficiency. Additional adverse events during the period of supplement use included cardiovascular and gastrointestinal complications. Of the 12 patients interviewed, 11 discontinued the supplement, of whom 8 stopped after recommendation from their physician.

Conclusion

Despite FDA warnings, patients with chronic musculoskeletal pain continue to use over-the-counter arthritis supplements due to community recommendation, perceived safety of the supplement, and limited relief from conventional therapies. Many are unaware of hidden pharmaceutical ingredients and experience serious adverse effects. Clinicians should routinely screen for supplement use, particularly in high-risk populations; provide clear, empathetic education about potential risks; and discuss safer pain management strategies.

## Introduction

Chronic pain affects nearly one in four Americans and remains a complex and widespread public health challenge [1]. One study estimates that 52% of people living with chronic nonmalignant pain turn to complementary and alternative medicine to manage their symptoms [2]. However, supplements often lack efficacy data, may interact with other medications, and may be contaminated with undisclosed heavy metals, toxic plant compounds, or prescription drugs [3-5]. Dietary and herbal supplements are not subject to pre-market FDA approval for safety or efficacy. If a post-market product is found to be unsafe or inappropriately labeled, the FDA can seek to restrict or remove the product through recalls, manufacturer warnings, and public notices [6,7].

In recent years, multiple over-the-counter arthritis pain supplements (Artri King, Atri Ajo King, Ortiga Ajo Rey, RM Flex, Advance King) advertised as containing glucosamine, chondroitin, collagen, and other ingredients have been found to contain hidden prescription drugs, namely dexamethasone, methocarbamol, and diclofenac. Several case studies and series have documented adverse effects associated with these hidden ingredients, including adrenal insufficiency, adrenal crisis, worsening glycemic control, osteoporotic fractures, immunosuppression, hyponatremia, and duodenal ulcers [8-17]. While the FDA has issued public warnings after receiving reports of adverse events from Artri King and related supplements [18], these products continue to be used in the community.

This study aimed to explore patients’ experiences with these arthritis pain supplements, focusing on motivations for use, adverse effects, and the influence of regulatory warnings.

This work was previously presented as a poster at the University of California Los Angeles Solomon Scholars Research Day on May 13, 2025.

## Materials and methods

This retrospective, mixed-methods cohort study was conducted at an urban, academic safety-net primary care clinic in California. Between April 2024 and March 2025, clinicians identified patients who reported the use of arthritis pain supplements reported by the FDA to contain hidden ingredients. Exclusion criterion was supplement use not known to contain hidden ingredients. Clinical data extracted from the electronic health record included demographics, medical history, presenting symptoms, presenting physical examination findings, relevant laboratory markers (e.g., morning cortisol, adrenocorticotropic hormone [ACTH], baseline and follow-up creatinine, baseline and follow-up hemoglobin A1c levels), subsequent corticosteroid use, and subsequent medical complications.

To complement the quantitative data, semi-structured telephone interviews were conducted to assess patient-reported motivations for supplement use, methods of acquisition, dosage and duration of use, understanding of the product's ingredients, and perceived benefits and adverse effects.

Patients provided verbal consent to participate in the structured phone interviews. This study was designated as exempt by the Olive View-University of California Los Angeles Education and Research Institute Institutional Review Board.

## Results

Clinicians identified 14 patients using arthritis pain supplements reported to contain hidden ingredients: Artri King (n=9, 64%), Artri Ajo King (n=4, 29%), and RM Flex (n=1, 7%) (Table 1). Patient ages ranged from 35 to 86 years, with equal representation of both sexes. Thirteen (93%) patients had a documented country of origin in Latin America; one (7%) patient was from India. Body mass index (BMI) ranged from 29 to 65, and eight (57%) patients carried a diagnosis of diabetes.

**Table 1:** Patient information and supplement use details *Declined interview. ^†^Did not recall supplement use. ^‡^Random cortisol (not AM cortisol). ^§^Tested during supplement use. ACTH, adrenocorticotropic hormone

Patient	Age	Sex	Supplement	Duration of use (months)	Pills/day	Side effects	Adverse events	Morning cortisol (mcg/dL)	Morning ACTH (pg/mL)	Discontinued (yes/no) + reason
1	78	F	Artri King	24	1	Weakness, non-healing wounds	Impaired wound healing, worsened diabetes control (after supplement cessation, A1c improved and patient no longer required antidiabetic medications)	Not tested	Not tested	Yes – physician advice
2*	64	M	Artri Ajo King	--	--	--	Gastroesophageal reflux disease	Not tested	Not tested	--
3	55	M	Artri King	24	2	Increased appetite	Three hospitalizations for decompensated heart failure, one hospitalization for fluid-resistant septic shock	58^‡^	9	Yes – television warning
4	54	M	Artri King	3	0.5	Weight gain	Duodenal ulcer	Not tested	Not tested	Yes – physician advice
5	43	M	Artri Ajo King	6	1	Mood changes, leg swelling	None	4.8	28	Yes – physician advice
6	51	M	Artri Ajo King	7	2	None	Adrenal insufficiency, persistent 3 months after cessation	<1	<5	Yes – physician advice
7	57	F	Artri King	2	2	Increased appetite, weight gain	Adrenal insufficiency, recovered after 7 months of cessation	<1	<5	Yes – physician advice
8	62	F	Artri King	0.2	1	None	None	Not tested	Not tested	Yes – physician advice
9	56	M	RM Flex	20	1-2	Easy bruising	Adrenal insufficiency, with ongoing supplement use	<1^§^	9	No – continued due to pain relief with supplement
10^†^	81	F	Artri King	--	--	--	Hypertension (resolved after supplement cessation), gastroesophageal reflux disease	Not tested	Not tested	--
11	81	M	Artri Ajo King	>60	3-6	Dizziness with supplement cessation	Presumed adrenal insufficiency, empirically treated with steroid replacement therapy	7.4^§^	Not tested	Yes – physician advice
12	86	F	Artri King	1	1	None	Worsening hypertension	Not tested	Not tested	Yes – physician advice
13	35	F	Artri King	2	1	None	None	Not tested	Not tested	Yes – pain ceased
14	76	F	Artri King	1	1	None	None	Not tested	Not tested	Yes – pain ceased

All 14 patients were contacted for a phone interview. One patient declined to participate, and another was unable to recall their supplement use. The remaining 12 (86%) consented to interview.

Motivation, acquisition, and use

All 12 interviewed patients reported using their supplement for musculoskeletal pain. Primary motivations included recommendations from friends or family (n=9, 75%), inadequate pain relief from provider-recommended treatments such as acetaminophen or topical/oral non-steroidal anti-inflammatory drugs (NSAIDs) (n=7, 58%), and the belief that the supplement was “natural” (n=5, 42%). Only one patient was familiar with the ingredients listed on their supplement's bottle. None of the patients were aware of the undisclosed ingredients (e.g., steroids, NSAIDs, or muscle relaxants), potential risks, or FDA warnings.

Patients reported acquiring the supplements abroad (n=3, 25%), at swap meets (n=3, 25%), or in small local stores (n=6, 50%). One patient (patient 9), who was later found to be adrenally insufficient, stated that only the RM Flex obtained abroad provided pain relief. By contrast, the US-purchased version, which the FDA only warns as containing diclofenac as a hidden ingredient, was ineffective.

Duration of use ranged from five days to over five years. Most patients reported taking one to two pills a day.

Benefits, side effects, and complications

All 12 (100%) interviewed patients reported significant pain relief from supplement use. Three (25%) patients noted improved energy and/or mobility; patient 11 was able to discontinue the use of a walker.

Seven (58%) patients reported side effects, all of whom had been using their supplement for at least two months. Side effects included weight gain or increased appetite (n=3, 25%), dizziness (n=1, 8%), weakness (n=1), leg swelling (n=1), mood changes (n=1), and non-healing wounds (n=1). Patients with mood changes and weight gain noted symptom onset within two weeks of initiation. In contrast, patient 1, who had poorly controlled diabetes, reported weakness and non-healing wounds after two years of supplement use.

Of the 14 patients whose charts were reviewed, morning cortisol levels were measured in five (36%) patients. Of these, three (60%) patients had low morning cortisol levels, suggestive of adrenal insufficiency (≤3 µg/dL), and two (40%) patients had equivocal results (3-16 µg/dL). No cosyntropin stimulation testing was performed. There was clinical evidence of adrenal insufficiency in two additional patients: one of the patients with an equivocal morning cortisol (patient 11) became dizzy after discontinuing his long-standing use of Artri King and was treated with fludrocortisone (0.2 mg daily) and a short taper of hydrocortisone (10 mg twice daily for two weeks and then 5 mg twice daily for two weeks). Another patient (patient 3) had a borderline morning ACTH level but no morning cortisol level and was hospitalized for fluid-resistant septic shock thought to be either due to adrenal insufficiency or acidosis from renal failure. He was treated with stress-dose steroids.

No other patients were treated with steroid replacement therapy. Among the three patients with low morning cortisol, two continued to show suppressed morning cortisol levels at four and six months following supplement cessation, while one recovered spontaneously after seven months.

Other complications noted through chart review included new or worsening hypertension (n=2, 14%), gastroesophageal reflux disease (n=2, 14%), recurrent hospitalizations for acute decompensated heart failure (n=1, 7%), duodenal ulcer (n=1, 7%), and worsening diabetes (n=1, 7%, with HbA1c improving from 11% to 5.5% after discontinuation of Artri King; insulin, metformin, and empagliflozin were all titrated off due to hypoglycemia).

Discontinuation of supplement use

Of the 12 interviewed patients, 11 discontinued supplement use by the time of the interview (92%), with 8 (67%) of these patients discontinuing after their physician's advice. Patient 1 initiated the discussion after noticing signs in the waiting room encouraging patients to speak with their primary care physician about supplement use. Only one interviewed patient, patient 9, continued supplement use despite medical advice, citing no other effective method of pain relief.

## Discussion

This retrospective, mixed-methods study builds on prior case reports and series documenting adverse events associated with over-the-counter arthritis supplements containing undisclosed drugs [8-17] while also exploring patient motivations and perceptions related to their use.

The majority of patients in our study began using these supplements based on recommendations from their community to manage chronic musculoskeletal pain. Many believed these supplements were "natural" and therefore safe. This belief - that herbal supplements are safer or carry fewer side effects due to their natural origin and over-the-counter availability - has been observed in other populations as well [19-20]. While many patients in this study cited limited relief from conventional treatments as a motivation for turning to alternative supplements, prior studies have found that dissatisfaction with traditional care may not serve as a consistent predictor of complementary medicine use [2,21].

Despite FDA warnings and the subsequent recall of these products from major retailers, none of the study participants were aware that the supplements contained undisclosed prescription medications. This underscores the challenge of educating patients in a regulatory environment where most supplements can be sold without being proven safe or effective, and action is only taken after harm occurs. Our findings also raise concerns about product variability across time and geographic location. While Artri King and similarly named products have been found to contain both dexamethasone and diclofenac, the FDA reported only diclofenac in RM Flex. Patient 9 developed adrenal insufficiency while using RM Flex sourced from outside the United States, suggesting that product composition may vary in ways that outpace FDA testing.

In our study, 75% of patients who were taking these supplements experienced side effects and/or clinical complications, similar to those reported in previous literature [8-17], with a trend toward more severe outcomes, such as symptomatic adrenal insufficiency or heart failure decompensation, among those with longer duration of use. Several interviewed patients had been taking the supplement for years before disclosing their use with their healthcare provider or choosing to discontinue. However, most were willing to stop use after learning about the potential harms.

These findings underscore the importance of regular, empathic inquiry by primary care providers and pharmacists about over-the-counter supplement use as part of a comprehensive medication reconciliation. A prior meta-analysis estimated that only around one-third of patients disclosed the use of complementary medicine to providers, with disclosure being more likely when providers inquire directly and when patients are aware of potential safety risks [22]. In populations where supplement use may be more common, such as our safety-net primary care population or the joint arthroplasty cohort described by Culler et al. [12], clinic signage may facilitate conversations about supplement safety. Given the widespread marketing of glucosamine and chondroitin for arthritis pain, clinicians should consider asking not only whether patients use these supplements but also which brand they use and where they obtained them.

This study has several limitations. First, the small sample size and retrospective design limit the generalizability of findings. The sample size further precludes statistical analysis. Second, as a retrospective study conducted at a single safety-net primary care clinic, findings may not reflect experiences in other populations or settings. Third, patient recall bias may have affected responses regarding duration, side effects, and supplement acquisition. Fourth, not all patients underwent hormonal testing and none underwent cosyntropin stimulation, limiting our ability to definitively diagnose or rule out adrenal insufficiency in many cases. Finally, selection bias may be present, as patients were identified through clinician reporting rather than systematic screening.

## Conclusions

Despite FDA warnings, patients with chronic musculoskeletal pain continue to use over-the-counter supplements containing hidden drugs such as Artri King, often under the impression that these products are safe and “natural." This study found that supplement use is largely driven by community recommendations, inadequate pain relief from traditional medical treatments, and belief that the supplement is safe. None of the participants were aware of the presence of hidden pharmaceutical ingredients or the potential for serious harm. While patients reported significant pain relief, many also experienced clinically significant side effects, including adrenal insufficiency, cardiovascular complications, and gastrointestinal disturbances.

This study highlights the urgent need for increased clinician awareness, proactive medication reconciliation, and patient education regarding the risks of unregulated supplements, especially in high-risk populations. Patients were often willing to discontinue these products once informed, reinforcing the importance of empathetic, informed conversations during routine care. Ultimately, ensuring safe and effective pain management depends not only on regulatory action but also on strong clinician-patient relationships and ongoing community education.
